# Identification of Lipotoxicity-Related Biomarkers in Diabetic Nephropathy Based on Bioinformatic Analysis

**DOI:** 10.1155/2024/5550812

**Published:** 2024-05-14

**Authors:** Han Nie, Huan Yang, Lidan Cheng, Jianxin Yu

**Affiliations:** Department of Endocrinology, Affiliated Hospital of Jiujiang University, No. 57, East Road, Xunyang District, Jiujiang, Jiangxi, China 332000

**Keywords:** bioinformatic analysis, biomarker, diabetic nephropathy, immune cell infiltration, lipotoxicity-related genes

## Abstract

**Objective:** This study is aimed at investigating diagnostic biomarkers associated with lipotoxicity and the molecular mechanisms underlying diabetic nephropathy (DN).

**Methods:** The GSE96804 dataset from the Gene Expression Omnibus (GEO) database was utilized to identify differentially expressed genes (DEGs) in DN patients. Gene Ontology (GO) and Kyoto Encyclopedia of Genes and Genomes (KEGG) enrichment analyses were conducted using the DEGs. A protein–protein interaction (PPI) network was established to identify key genes linked to lipotoxicity in DN. Immune infiltration analysis was employed to identify immune cells with differential expression in DN and to assess the correlation between these immune cells and lipotoxicity-related hub genes. The findings were validated using the external dataset GSE104954. ROC analysis was performed to assess the diagnostic performance of the hub genes. The Gene set enrichment analysis (GSEA) enrichment method was utilized to analyze the key genes associated with lipotoxicity as mentioned above.

**Result:** In this study, a total of 544 DEGs were identified. Among them, extracellular matrix (ECM), fatty acid metabolism, AGE-RAGE, and PI3K-Akt signaling pathways were significantly enriched. Combining the PPI network and lipotoxicity-related genes (LRGS), LUM and ALB were identified as lipotoxicity-related diagnostic biomarkers for DN. ROC analysis showed that the AUC values for LUM and ALB were 0.882 and 0.885, respectively. The AUC values for LUM and ALB validated in external datasets were 0.98 and 0.82, respectively. Immune infiltration analysis revealed significant changes in various immune cells during disease progression. Macrophages M2, mast cells activated, and neutrophils were significantly associated with all lipotoxicity-related hub genes. These key genes were enriched in fatty acid metabolism and extracellular matrix-related pathways.

**Conclusion:** The identified lipotoxicity-related hub genes provide a deeper understanding of the development mechanisms of DN, potentially offering new theoretical foundations for the development of diagnostic biomarkers and therapeutic targets related to lipotoxicity in DN.

## 1. Introduction

Diabetes is a prevalent global health issue, with an estimated 439 million people projected to have diabetes by 2030 [1]. Diabetic nephropathy (DN) is the most common complication of diabetes, leading to end-stage renal failure and cardiovascular events [2, 3]. About 40% of diabetes patients develop type 2 diabetes within 10 years of diagnosis [4]. DN is characterized by persistent albuminuria (excretion rate > 300 mg/d or 200 *μ*g/min), requiring at least two measurements within 3 to 6 months to confirm, along with a gradual decline in renal function (GFR) [5]. Chronic hyperglycemia and hypertension are primary risk factors for DN, although the exact pathogenesis remains incompletely understood. Current main treatments include renin–angiotensin system blockers, anti-inflammatory agents, and antioxidative stress measures [2]. Despite these treatments, there is still a significant risk of disease progression, and the use of microalbuminuria as an early diagnostic marker for diabetic kidney disease (DKD) has limitations. Some diabetic patients may develop DKD even with normal urinary albumin levels [6, 7], underscoring the importance of identifying new biomarkers and exploring the molecular mechanisms of DKD.

Lipotoxicity is a condition characterized by the abnormal accumulation of lipids in nonadipose tissues, disrupting cellular balance and leading to metabolic, inflammatory, and oxidative stress (OS) responses that ultimately result in cell death [8]. This lipid buildup, particularly in the kidneys, is closely linked to the development of kidney diseases such as DN [9]. Research suggests that disturbances in lipid quantity and quality play a crucial role in the renal damage caused by lipotoxicity [9]. The excessive accumulation of lipids induces insulin resistance, activating lipid synthesis and glycogen pathways. Elevated levels of esterified fatty acids (NEFAs) contribute to podocyte injury, renal tubular damage, mesangial cell proliferation, endothelial activation, and the formation of foam cells derived from macrophages [10]. Lipotoxic effects impact various renal cell types, with lipid deposits surrounding glomeruli and tubules, leading to glomerular enlargement and tubulointerstitial fibrosis [11]. The rise in saturated free fatty acids, particularly palmitic acid (PA) and steroidal acid, is considered a key factor in the damage to proximal tubular cells and podocytes in DN. Therefore, safeguarding renal cells from lipotoxicity associated with saturated free fatty acids may present a promising therapeutic strategy for patients with challenging cases of DN [12]. Sirtuins, a group of NAD-dependent deacetylase enzymes, particularly SIRT3, have been identified as potential mitigators of lipotoxicity-induced tubular injury. In kidneys affected by FFA-binding proteinuria, the decrease in SIRT3 expression is linked to increased MCP-1 expression. Augmented SIRT3 levels help lower reactive oxygen species accumulation by boosting the oxidative capability of proximal tubular epithelial cells when exposed to PA [13]. This suggests that reduced SIRT3 expression might contribute to the development of lipotoxicity-induced renal tubular cell injury and could serve as a promising target for therapy to prevent and manage DN progression. Sterol regulatory element binding protein (SREBP) plays a crucial role in lipid metabolism alterations, as evidenced in DN studies [14]. SREBP-1 is a pivotal player in promoting lipid buildup in renal tubular damage and inflammation in DKD [15]. While some research indicates its association with TGF-*β* activation leading to worsened tubulointerstitial fibrosis [16], the exact effects and mechanisms of DN necessitate further investigation. FFA-triggered DAG-PKC pathway activation sparks inflammatory responses in cultured proximal tubular cells by upregulating NF*κ*B and causing mitochondrial cell death [17]. Palmitate triggers TLR4 signaling and inflammation through downstream pathways in proximal tubular cells [18]. Kidney-protective effects are seen with PPAR*α* agonists, and the PPAR*δ* agonist GW501516 can curb NF*κ*B-mediated MCP-1 overexpression by reducing TLR4/TAK1 activity [18].

The PPAR*α* agonist fenofibrate tablets inhibit palmitate-induced proximal tubular cell damage by enhancing fatty acid oxidation and reducing saturated FFA-related lipotoxicity in mice and cultured proximal tubular cells [19]. Research has also shown that inhibiting the TLR4 signaling pathway can eliminate proinflammatory cytokine synthesis induced by saturated FFA, indicating a potential renoprotective effect in diabetic podocyte pathology. Studies have demonstrated that palmitate can activate mTORC1, leading to podocyte damage and apoptosis [20], thus establishing a link between mTORC1 activation and podocyte damage in patients with diabetes. Currently, the majority of research on lipotoxicity is centered around fatty liver, with clinical intervention drugs primarily focused on preventing nonalcoholic fatty liver. Limited attention has been given to the kidneys, particularly DN, and the underlying mechanisms in DN remain largely unknown. This area of study is still in its early stages and requires further investigation. To address this gap, the present study utilized network bioinformatics data mining techniques to analyze differentially expressed genes (DEGs) in DN patients. Enrichment analysis was conducted to identify relevant biological pathways, and a protein–protein interaction (PPI) network was established using DEGs to pinpoint key genes. By intersecting these key genes with lipotoxicity-related genes (LRGS), important LRGS were identified. Additionally, immune infiltration analysis revealed changes and correlations among different immune cells, along with an analysis of the relationship between immune cells and key LRGS. The primary objective of this study is to uncover crucial LRGS that play a significant role in DN progression and their influence on the immune microenvironment. Ultimately, this research is aimed at offering novel targets for the prevention and treatment of DN.

## 2. Materials and Methods

### 2.1. Microarray Data and Preprocessing

**Figure 1:**
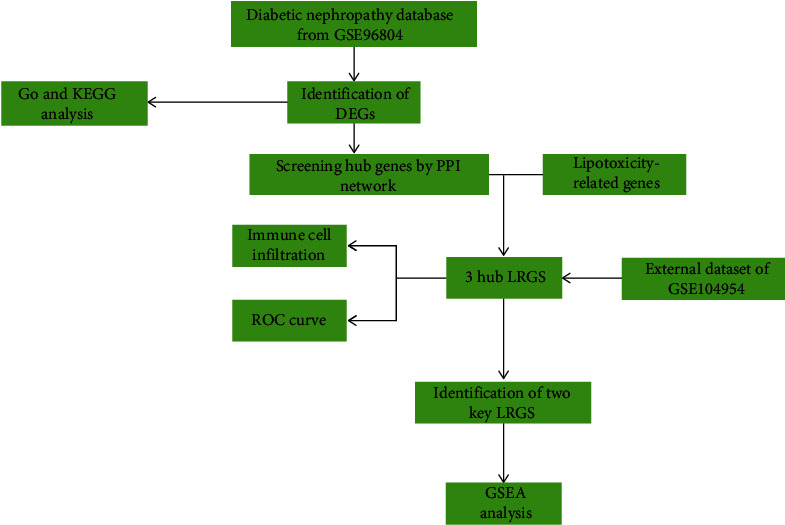
Flow chart of the study. DEGs: differentially expressed genes; LRGS: lipotoxicity-related genes.

Expression profiling by array was searched in the Gene Expression Omnibus (GEO) database using the keywords DN, DKD, and *Homo sapiens*. It was decided to screen and download the gene expression microarray datasets: GSE96804 (GPL17586 Affymetrix Human Transcriptome Array 2.0) contained forty-one cases of DNs and twenty controls, and GSE104954 (GPL24120 Affymetrix Human Genome U133A Array) contained ten DNs and five control samples, respectively. Additionally, we use a relevance score of greater than 0 as a screening criterion in order to extract 433 genes associated with lipotoxicity from the GeneCard database and online literature [21]. The details of the above datasets are shown in Table 1. The flow chart of the research is shown in Figure 1.

### 2.2. Identification of DEGs

After normalization and preprocessing of the data, the probes were annotated. It was done through the “GEOquery” package of R software. If multiple probes correspond to the same gene, only the probe with the highest average expression is retained. After completion of probe annotation with |log^2^ fold change (*FC*)| > 1 and *p* < 0.05 as screening criteria, DEGs from GSE96804 were identified utilizing “Limma” R package, where log FC > 1, *p* < 0.05 was up and log FC ≤ 1, *p* < 0.05 was down. The heat map and volcano map of DEGs were plotted using the “Pheatmap” R package and “ggplot2” R package, respectively.

### 2.3. Functional Annotation and Enrichment Analysis of DEGs

To further illuminate the biofunction of the selected DEGs, Gene Ontology (GO) enrichment analysis and Kyoto Encyclopedia of Genes and Genomes (KEGG) pathway analysis were done using “ClusterProfiler” (version 4.6.2) within R on DEGS. And then the “ggplot2” R package was used to screen the biological process (BP), cellular component (CC), molecular function (MF), gene-related signaling pathways, and *p* value < 0.05 as a threshold.

### 2.4. Construction of PPI Network and Identification of Hub Genes

Import the DEGs into the STRING database to construct the PPI network. It was visualized by importing the results of the analysis into Cytoscape software. The core targets of the PPI network were analyzed using the CytoHubba plug-in. The top 10 targets with MCC, MNC, degree, and closeness scores were calculated separately [22]. The intersection of the four scores was hub genes. The results were screened using the Venn diagram.

### 2.5. CIBERSORT Immune Cell Infiltration Analysis

We used the CIBERSORT method to identify the ratios of 22 immune cell species in all samples in the DN and control groups and to depict the abundance of immune cells based on the gene expression matrix. The “corrplot” program was allowed to construct the heat map illustrating the quantitative relationship between the different immune cells, and *p* value < 0.05 indicates a statistically significant difference. In addition, the “ggplot2” program package was employed to analyze the correlation between the expression of hub LRGs and the proportions of immune cells.

### 2.6. Construction and Validation of a Nomogram

The R package “ggplot2” (version 4.2.1) was used to calculate the expression of hub LRGS in the DN and control groups. The area under the receiver operating characteristic (ROC) curve (AUC) was recognized as the quantitative evaluation criterion for determining the discrimination capacity of each hub LRG. The ROC analysis was performed using the R package “pROC” (version 1.18.0) [23].

### 2.7. GSEA

In addition, a signal gene set enrichment analysis (GSEA) was utilized to determine the most significant roles of hub LRGS [24]. The predefined gene set was obtained from the C2 KEGG database. These gene sets summarize and represent well-defined signal pathways and have consistent expression. One thousand times gene set permutations were performed in order to obtain a normalized enrichment score in each analysis. After 1000 permutations, a false discovery rate (FDR) < 0.25 and *p* value < 0.05 were considered highly enriched.

### 2.8. Statistical Analysis

The Wilcoxon rank-sum test was used to verify statistical significance comparisons between groups in nonnormally distributed data. Correlation scores were calculated using Spearman's method. *p* < 0.05 was considered statistically different.

## 3. Results

The specific study chart is shown in Figure 1.

### 3.1. Screening DEGs

**Figure 2:**
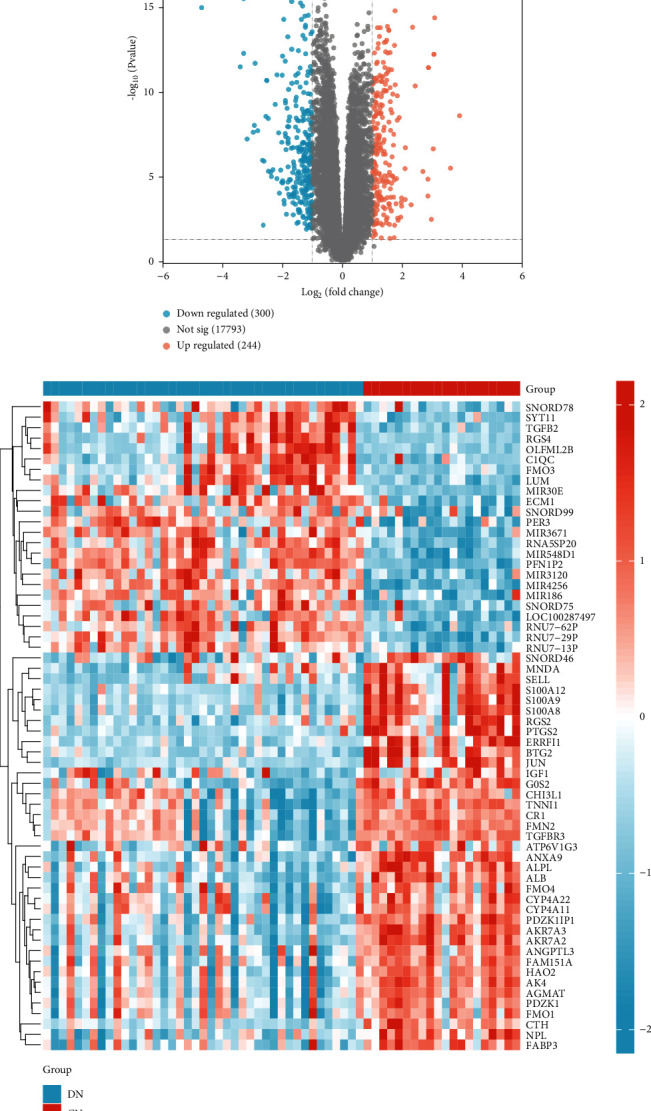
Identification of DEGs in GSE96804 (a) volcano plot of DEGs and (b) heat map of DEGs.

According to the set screening criteria (*p* < 0.05 and |log *FC*| > 1), a total of 544 DEGs were obtained after screening. Among them, there were 244 upregulated DEGs and 300 downregulated DEGs. The volcano plot and heat map of DEGs distribution are shown in Figures 2(a) and 2(b)).

### 3.2. Biological Pathway Enrichment Analysis

**Figure 3:**
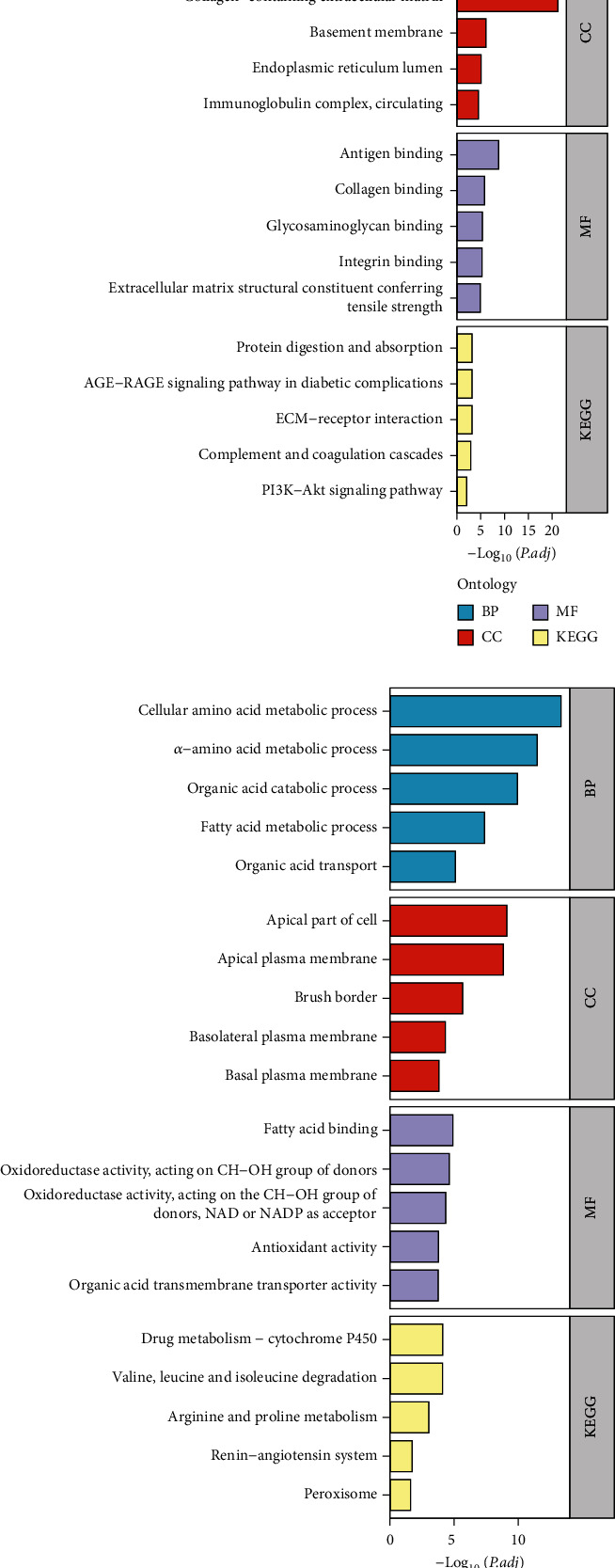
Functional enrichment analysis of DEGs. (a) Results of GO and KEGG in upregulated DEGs are depicted on bar charts. (b) Results of GO and KEGG in downregulated DEGs are depicted on bar charts. GO: Gene Ontology; KEGG: Kyoto Encyclopedia of Genes and Genomes; BP: biological process; CC: cellular component; MF: molecular function.

The GO and KEGG pathway enrichment analyses were performed using upregulated/downregulated DEGs separately. In the BP assessment, upregulated DEGs were mostly involved in complement activation, production of molecular immune responses, and other pathways that may be activated. The DEGs have been localized to the collagen-containing extracellular matrix (ECM), basement membrane, and other structures in CC. The function changes of DEGs are associated with antigen binding and collagen binding. According to KEGG analysis, DEGs were mostly engaged in the AGE-RAGE signaling pathway, ECM-receptor interaction, and PI3K-Akt signaling pathway (Figure 3(a)). In the enrichment results of downregulated DEGs, DEGs were engaged mostly in the organic acid process, fatty acid process, fatty acid binding, and other processes in BP. The DEGs have been localized to the apical part of the cell, apical plasma membrane, and brush border in CC. In terms of MF, DEGs were associated with fatty acid binding, antioxidant activity, and organic acid transmembrane transporter activity. According to KEGG analysis, DEGs were particularly abundant in drug metabolism, renin–angiotensin system, arginine and proline metabolism, and other pathways (Figure 3(b)).

### 3.3. Construction of PPI Network and Identification of Hub Genes

**Figure 4:**
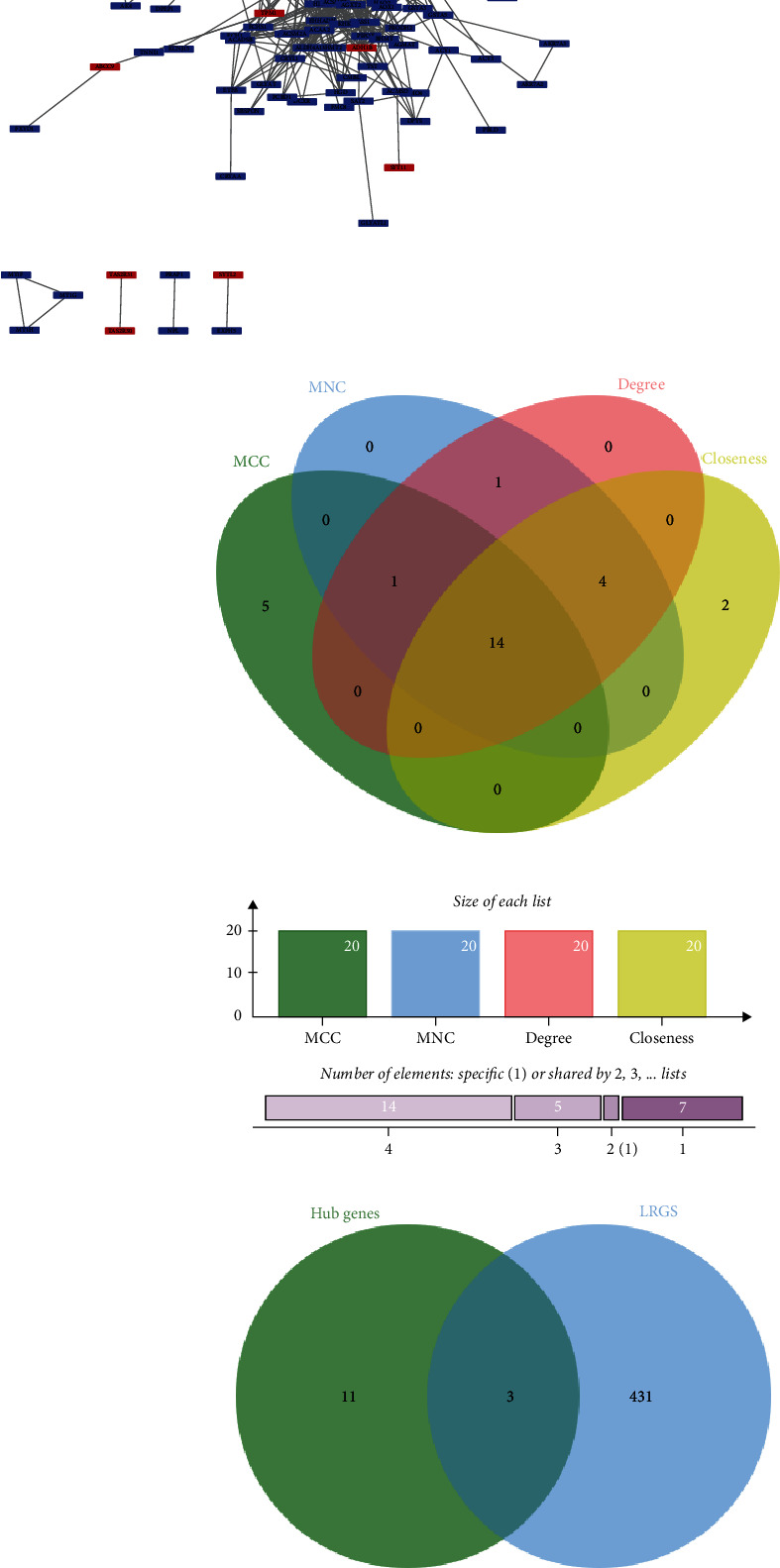
Construction of PPI networks and identification of hub genes and hub LRGS. (a) PPI network constructed using DEGs. Each diamond represents a DEG. (b) The hub genes were screened by four algorithms (MCC, MNC, degree, and closeness). (c) Intersecting hub genes with LRGS to identify hub LRGS. PPI: protein–protein interaction.

PPI networks are created using the STRING database. The generated network has 371 nodes, 1727 edges, an average node degree was 9.31, and PPI enrichment *p* value < 1.0e − 16. The results were imported into Cytoscape software for visualization (Figure 4(a)). Using the CytoHubba plug-in to calculate MNC, MCC, degree, and closeness scores and taking their intersection, a total of 15 hub genes were found: FN1, MMP2, COL3A1, COL1A2, POSTN, LUM, LOX, IGF1, CCL2, PTGS2, EGF, ALB, JUN, and KDR (Figure 4(b)). A total of 433 LRGS were obtained from previous studies, and a total of 3 hub LRGS were obtained by intersecting LRGS with hub genes: LUM, IGF1, and ALB (Figure 4(c)).

### 3.4. Immune Infiltration Analysis

**Figure 5:**
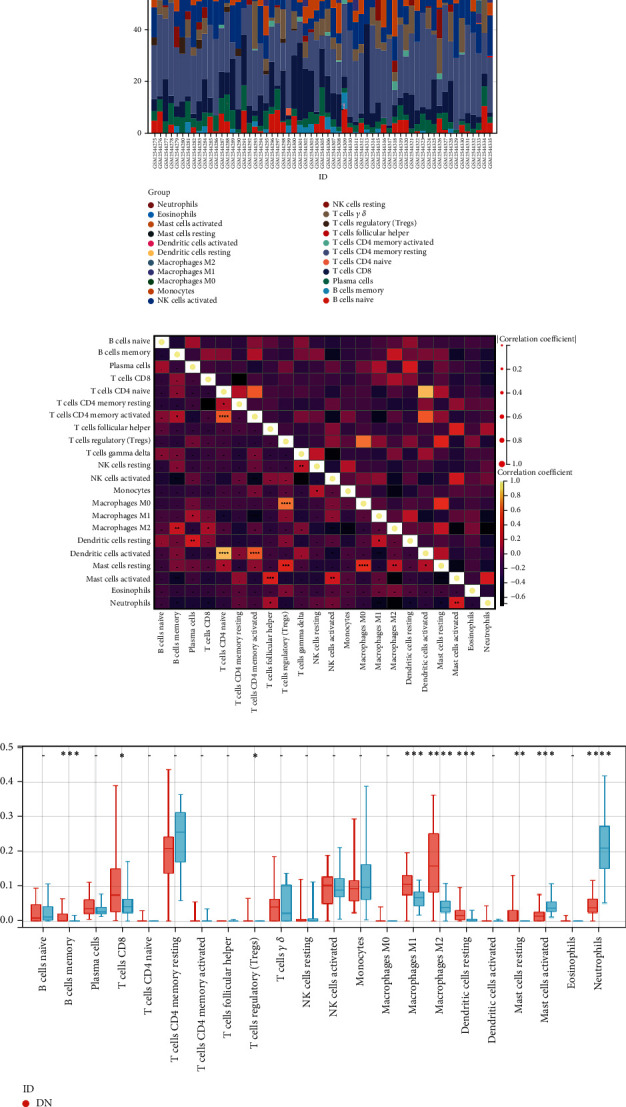
Immune cell infiltration analysis. (a) Distribution of 22 kinds of immune cells in tissues of the DN and control groups. (b) Correlation diagram between immune cells. (c) Expression of immune cells in the DN and control groups.

We measured the characteristics of immunocytes through the CIBERSORT algorithm to further explore the differential expression of immune components in each sample, and the relative proportions of 22 immune cells were displayed in the cumulative histograms (Figure 5(a)). The results indicated that T cells CD8, T cells CD4 naive, T cells CD4 memory activated, T cells regulatory (Tregs), monocytes, macrophages MO, macrophages M2, and mast cells resting constituted the majority. The correlation heat map for 22 immune cell types revealed a strong positive link between T cells CD4 naive and dendritic cells activated, T cells CD4 naive and T cells CD4 memory, T cells CD4 memory activated and Tregs, and macrophages MO and Tregs and a significant negative correlation between T cells CD4 memory resting and T cells CD8, NK cells activated and T cells gamma delta, macrophages M2 and mast cells activated, and macrophages M2 and neutrophils (Figure 5(b)). In addition, the box plot revealed significantly higher T cells CD8, Tregs, macrophages M2, macrophages M1, and dendritic cells resting in the DN group than in the CN group, whereas mast cells resting, mast cells activated, and neutrophils were significantly decreased (Figure 5(c)).

### 3.5. Correlation Analysis of Immune Cells and Hub LRGS

**Figure 6:**
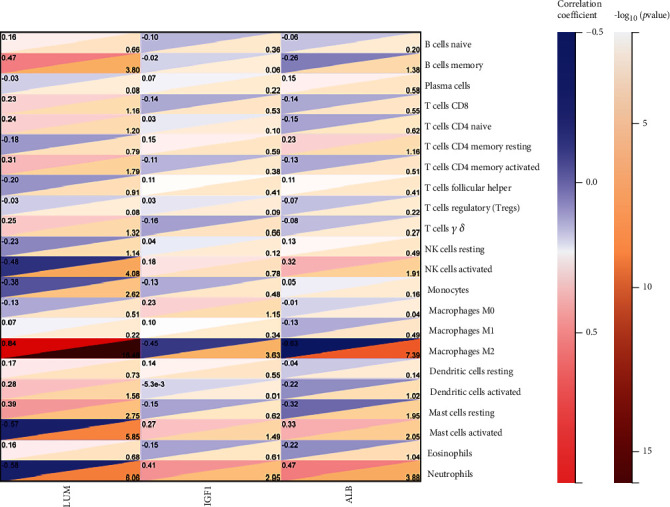
Correlation of immune cells with hub LRGS.

We used the Spearman correlation coefficient between hub LRGS and immune cells to further explore the correlation between hub LRGS and immune cells and visualize it in the correlation heat map (Figure 6). The results showed that the immune cells that were significantly correlated with three hub LRGs were macrophages M2, mast cells activated, and neutrophils. It implicated a significant influence on these immune cells during the regulation of hub LRGs in disease. They may play a regulatory role in the advancement of DN.

### 3.6. Expression of Hub LRGS and Validation of External Datasets

**Figure 7:**
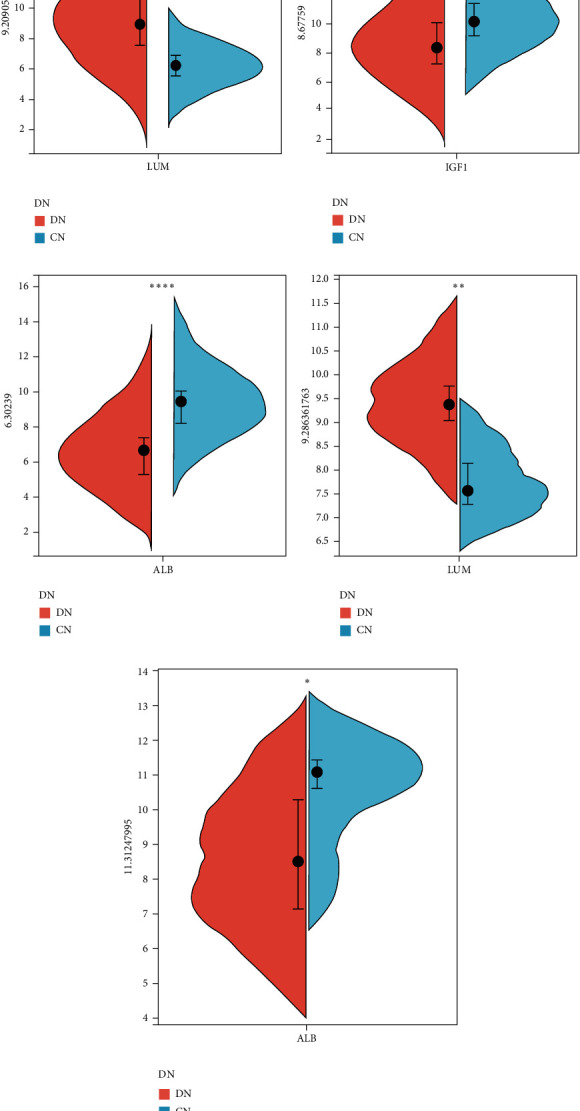
Split violin plot of the expression of hub LRGS in different datasets. The expression of hub LRGS in the GSE96804 dataset is shown in Figures 6(a)–6(c)). The expression of hub LRGS in the GSE104954 dataset is shown in Figures 6(d) and 6(e)).

We discovered in the GSE96804 datasets that the expression levels of LUM and IGF1 were higher in DN (Figures 7(a) and 7(b)), while ALB expression was significantly lower in DN than in control (Figure 7(c)). We next confirmed the expression of these genes using another dataset, and the results revealed that LUM and ALB expression levels were found to be consistent in the tubulointerstitial (GSE104954), and they were all statistically significant differences (Figures 7(d) and 7(e)). IGF1 was not found in the GSE104954 dataset.

### 3.7. ROC Curve Analysis

**Figure 8:**
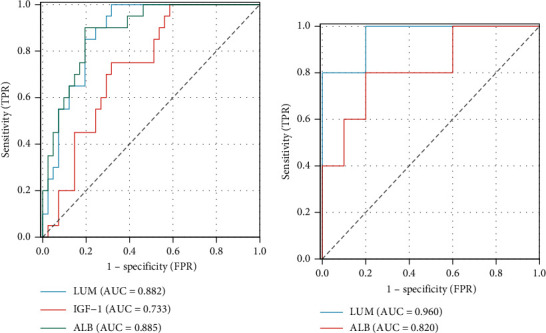
ROC curve analysis. (a) Hub LRGS in the GSE96804 datasets were analyzed using ROC curves. (b) Hub LRGS in the GSE10495 datasets were analyzed using ROC curves.

A ROC curve analysis was implemented to test the diagnostic value of the hub LRGS, and an AUC value > 0.7 in these hub LRGS was used as diagnostic criteria. In the GSE96804 datasets, the AUC values were 0.882 for LUM and 0.885 for ALB (Figure 8(a)). In the GSE10495 datasets, the AUC value of LUM was 0.98 and the AUC value of ALB was 0.82 (Figure 8(b)).

### 3.8. GSEA

**Figure 9:**
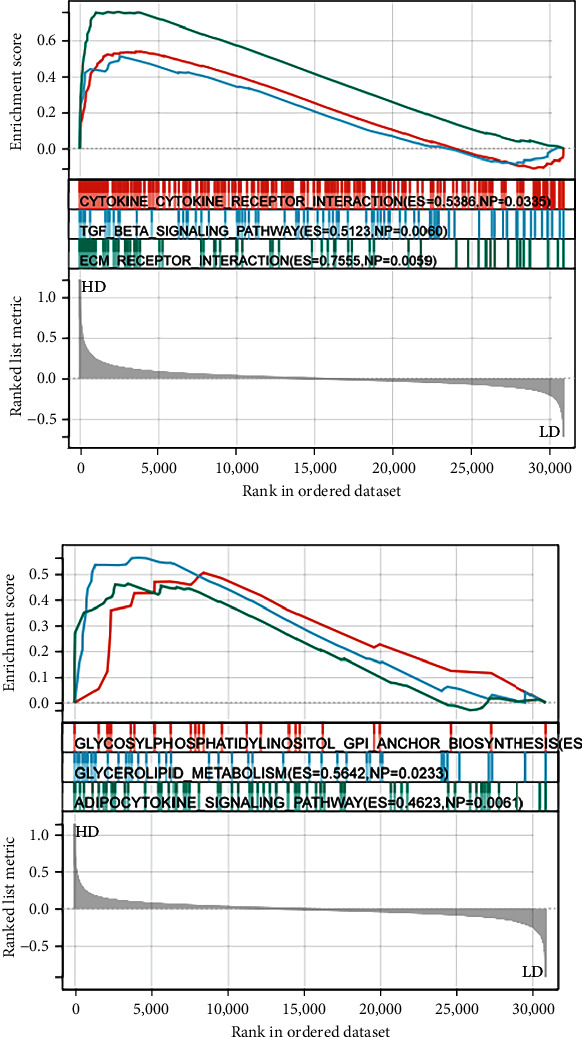
(a, b) GSEA of hub LRGS.

The result of GSEA shows that the LUM high expression group was highly enriched for cytokine–cytokine receptor interaction, TGF-*β* signaling pathway, and ECM receptor interaction (Figure 9(a)). The ALB high expression group was mostly involved in glycosylphosphate, metabolism, and adipocytokine signaling pathway (Figure 9(b)).

## 4. Discussion

DN is a complex condition influenced by multiple factors, although its exact mechanism remains unclear and current treatment options are limited. Recent research has identified new molecules for both treating and diagnosing DN. Studies suggest that the progression of DN is linked to both lipotoxicity and immunity. Enrichment analysis has highlighted the significance of pathways such as AGE-RAGE signaling, ECM receptor interaction, TGF-*β* signaling, PI3K-Akt signaling, and fatty acid metabolism in relation to lipotoxicity. A PPI network was constructed, and central genes related to lipotoxicity were identified through various analysis methods. Two key genes, lumican and ALB, showed promising diagnostic value in ROC curve analysis. Furthermore, the CIBERSORT algorithm revealed potential correlations between these genes and immune cells, suggesting a role in regulating the immune microenvironment in DN.

KEGG enrichment analysis was performed separately on upregulated and downregulated DEGs. The enrichment analysis results of upregulated DEGs mainly focused on the AGE-RAGE signaling pathway, ECM-receptor interaction, and PI3K-Akt signaling pathway [25]. High glucose induces the formation of advanced glycation end products (AGE) and their receptors (RAGEs), altering various intracellular signaling mechanisms and leading to the occurrence and progression of DN [25]. The RAGE was initially described as a signal-transduction receptor for AGEs and is also considered a signal-transduction receptor associated with proteins or lipids [26]. AGEs belong to nonenzymatic glycation and are products of protein and lipid oxidation accumulated in diabetes and various inflammatory lesions [27, 28]. Once bound to RAGE, they can regulate lipid metabolism [29]. An animal study found 31 significantly altered lipid metabolism products in a DN mouse model, including glycerophospholipid metabolism and sphingolipid metabolism, which can participate in the development of DN by regulating the AGE-RAGE and PI3K/Akt signaling pathways affecting insulin resistance and abnormal lipid accumulation [30]. Excessive lipid flux and biologically active lipid substances, such as triglycerides (TG), ceramides, and other derivatives, not only release saturated free fatty acid signals but also enhance the input of free fatty acids into the triglyceride pool. Additionally, they directly counteract insulin signal transduction, leading to insulin resistance [31]. Elevated plasma NEFA concentrations are recognized as an autonomous risk factor for insulin resistance. Prolonged exposure to NEFA raises levels through two distinct mechanisms: metabolic interference leading to cell death, reduced insulin secretion, direct downregulation of insulin transcription, and beta cell death [20]. FFA activates G protein-coupled receptor 40 (also known as FFAR1), and single nucleotide polymorphisms in the FFAR1 locus are associated with human insulin secretory function. FFAR1 agonists, such as Tak875 and Gw-9508, enhance glucose-stimulated insulin secretion in diabetic ZDF rats, leading to positive effects on insulin secretion. Saturated fatty acids reduce FFAR1 expression and induce insulin resistance, potentially contributing to lipotoxicity. Studies have shown that treatment with palmitate on human pancreatic islet tissue decreases insulin content and secretion, but this adverse outcome can be reversed by FFAR1 antagonists [17].

Dyslipidemia encompasses various abnormal blood lipid levels, including low-density lipoprotein (LDL-R), free fatty acids, abnormal lipoproteins, ceramides, and other lipids. These components can impact the proximal renal tubules by increasing the release of reactive oxygen species and lipid peroxidation, leading to inflammation and mitochondrial damage in epithelial cells, podocytes, and renal tubulointerstitial tissues. The AGE/RAGE signaling pathway has been identified as a key target of lipid action, with certain drugs targeting these pathways showing potential in improving symptoms of DN [32]. For instance, using different concentrations of the AGE/ALE inhibitor LR-90 in STZ diabetic rats' kidneys has been found to reduce renal AGE/ALE accumulation and RAGE protein expression in a concentration-dependent manner, thereby preventing lipid peroxidation, lowering blood lipid levels, and mitigating the progression of diabetic renal disease [33]. Additionally, HMG-R inhibitor statins have shown promise in inhibiting the interaction of glycation end products (AGEs) with receptors for glycation end products (RAGE) and sugar-oxidized LDL-R, thereby reducing LDL receptor (LDL-R) uptake of LDL. This regulation of cholesterol synthesis through HMG-R helps reduce abnormal lipid deposition in the kidney [34], potentially offering a protective effect against DN. Ezetimibe (EZ) significantly reduces renal AGE levels in diabetic rats and downregulates renal AGE receptors, decreases lipid peroxidation and protein-bound carbonyl content (CC), increases paraoxonase-1 (PON-1) associated with high-density lipoprotein (HDL) and renal antioxidant enzyme activity, improves lipid and lipoprotein levels in the body, and protects the kidneys [35]. Abnormal lipid metabolism plays an important role in the pathogenesis of DN, and Akt activation has been shown to be associated with fat synthesis [36]. Overexpression of sterol regulatory element-binding protein-1 (SREBP-1) mediates abnormal lipid accumulation in DN renal tubular epithelial cells, and inhibiting the PI3K/Akt pathway can reduce SREBP-1 expression and lipid accumulation, indicating that the PI3K/Akt signaling pathway plays an important role in mediating high glucose-induced SREBP-1 expression in DN renal tubular cells [37]. Another animal and cell experiment also found that inhibiting the mTOR/AMPK/PI3K/Akt signaling pathway in DN can significantly reduce levels of total cholesterol (TC), TG, and low-density lipoprotein (LDL-C) while increasing HDL-C levels and improving blood lipid abnormalities [38]. Various drugs that regulate metabolic disorders have been shown to reduce blood sugar levels in diabetic rats by activating the PI3K/Akt pathway and upregulating PPAR*γ* expression. This results in improved insulin resistance, blood lipid profile levels, reduced adipocyte inflammation, and potential benefits for diabetic microvasculature [39, 40]. Further research is needed to determine the impact of the PI3K/Akt pathway on lipid metabolism in DN. The accumulation of ECM protein in the glomerulus is a key factor in the progression of DN to end-stage renal failure [41]. Transforming growth factor beta (TGF-*β*) plays a crucial role in this process by upregulating genes encoding ECM proteins and downregulating genes for ECM-degrading enzymes [42]. In a high-glucose environment, polyols and hexosamines increase, leading to the production of reactive oxygen species, activation of the TGF-*β*-Smad-MAPK pathway, increased production of AGEs, and stimulation of renal function. TGF-*β*1 produced by glomerular cells contributes to glomerulosclerosis and excessive ECM accumulation, resulting in tubulointerstitial fibrosis damage, cellular dysfunction, and progression of DN [43]. Abnormal accumulation of ECM not only plays a crucial role in renal fibrosis in DN but also impacts lipid metabolism. Lipotoxicity in nonadipose tissues caused by hyperlipidemia and lipid peroxidation contributes to the progression of DN, with a connection to lipid oxidation. OS is linked to fibrotic lesions [44]. Research by Yang et al. demonstrated that obese rats with TLR4 gene knockout could attenuate PA-induced lipotoxicity in islet *β* cells and improve insulin secretion disorders through the ECM-receptor pathway, highlighting the significance of this pathway in lipotoxicity [45]. Additionally, a study on nonalcoholic fatty liver disease revealed that diabetic mice with reduced insulin receptor (InsR) fed a methionine choline (MCD)-deficient diet exhibited impaired liver fat content and hepatic insulin signaling, leading to the accumulation of Forkhead box protein O1 (FoxO1) and subsequent induction of lysyl oxidase-like 2 (Loxl2). This enhanced liver cell lipotoxicity induced insulin resistance and abnormal ECM deposition and ultimately resulted in liver fibrosis [46], illustrating the intricate relationship between ECM and cellular lipotoxicity. Enrichment analysis of downregulated differential genes revealed their association with the renin–angiotensin system, a pivotal factor in the pathogenesis of DN. This system has been extensively linked to the development of DN and serves as a cornerstone of its management. Various drugs targeting the renin–angiotensin system, including sodium-glucose cotransporter 2 inhibitors, steroidal mineralocorticoid receptor antagonists [47], and glucagon GLP-1 receptor agonists, have been investigated for their potential to slow disease progression and exhibit cardiorenal protective effects [48]. GO enrichment analysis suggests a potential involvement of fatty acid metabolism in the pathogenesis of DN. Elevated levels of saturated free fatty acids are recognized as contributors to proximal tubular and podocyte damage in this condition [49]. Disorders in fatty acid oxidation and lipid metabolite accumulation are also significant factors leading to kidney tissue damage. Studies have identified a substantial presence of lipid deposits and intracellular accumulation in the kidneys of individuals with DN, accompanied by increased lipid droplets and downregulation of fatty acid *β*-oxidation pathways such as L-FABP. These findings support the hypothesis that reduced fatty acid oxidation contributes to abnormal lipid accumulation [9]. Another study on metabolomics in patients with DKD revealed elevated levels of lipid metabolites LPC (16:0) and (18:0) in the renal tubulointerstitium, which were linked to a rapid decline in renal function. Subsequent animal and in vitro experiments confirmed these results, suggesting a role for lipid metabolites in mediating lipotoxicity in proximal renal tubular cells [50]. In contrast, lipotoxicity in glomerular podocytes is characterized by increased fatty acid uptake, enhanced synthesis, or reduced degradation. Podocyte-specific JAML upregulation was found to increase the expression of sirtuin-1-dependent cholesterol regulatory element binding protein (SREBP)-1, facilitating free fatty acid synthesis. Furthermore, upregulation of the G protein-coupled FFA1 receptor and CD36 scavenger receptor was observed, promoting fatty acid uptake and leading to fatty acid accumulation in podocytes [51]. The results of the GO and KEGG enrichment analyses underscore the significance of lipotoxicity in the progression of DN.

To further explore the role of lipotoxicity in DN, we utilized differential gene analysis to construct a PPI network diagram, identifying 15 hub genes. These hub genes were then intersected with lipotoxicity-related disease genes, leading to the identification of three key LRGS: lumican, ALB, and IGF1. Subsequent validation with external datasets confirmed that lumican and ALB are crucial LRGS with significant diagnostic potential. Lumican, a biologically active 40 kDa member of the keratin sulfate proteoglycan family, is primarily expressed in the lung [52], cornea [53], skeletal muscle [54], and liver tissues. Proteomic analysis by Charlton et al. revealed lumican protein overexpression in mild and fibrotic nonalcoholic fatty liver disease, suggesting a novel mechanism of lipotoxicity involving decreased free fatty acid-binding proteins and impaired free fatty acid clearance [55]. As a component of the ECM, lumican plays a role in collagen fiber formation, interacts with transforming growth factor-*β*1 (TGF-*β*1), and contributes to renal cell death in DN by promoting glomerulosclerosis and renal tubular fibrosis [56, 57], aligning with our enrichment analysis findings. A study on liver fibrosis demonstrated that lumican can be upregulated by the profibrotic cytokine TGF-*β*1 and lipotoxic PA [58]. This upregulation leads to increased collagen fiber production through the secretion of collagen in subcutaneous adipose tissue, exacerbating abnormal cellular lipid metabolism and inducing insulin resistance [59]. Lumican has been associated with adipocyte dysfunction and its impact on fat metabolism in diabetic patients. In animal and model studies of diabetes mellitus (DM), treatment with human recombinant lumican has been shown to enhance lipolysis in adipocytes and elevate free fatty acid levels in the body [60]. While most research on lumican lipotoxicity has focused on nonalcoholic fatty liver disease, further investigation is needed to elucidate its specific mechanism of action in the pathogenesis of DN.

Human serum albumin (ALB), a plasma albumin synthesized in the liver, has the ability to bind various substances and is increasingly being recognized as a potential predictive marker for certain diseases. While proteinuria and glomerulopathy are known risk factors for DN, the impact of serum albumin levels on kidney function remains unclear. Zhang et al. conducted a study on patients with DN and observed that hypoalbuminemia in diabetic patients with renal disease is linked to a poorer prognosis and a higher risk of developing end-stage renal disease, independent of proteinuria levels, indicating the involvement of other pathogenic mechanisms [61]. As DN progresses, massive proteinuria is often accompanied by a decrease in serum albumin levels. A prospective study revealed a positive correlation between massive proteinuria in DN patients and serum adiponectin, which can potentially predict the severity of DN [62]. It is suggested that albumin may be linked to the adipocytokine signaling pathway, consistent with the findings of this study's enrichment analysis. Albumin isolated from DN patients undergoes significant aminoacylation, which can hinder cholesterol efflux mediated by HDL2 and HDL3, leading to abnormal lipid accumulation [63]. In a state of disrupted blood glucose and lipid metabolism, filtered albumin exposes albumin-bound long-chain fatty acids on the surface of the proximal tubule. Notably, albumin itself does not exhibit cytotoxicity to the proximal tubule [64, 65]. It has been shown that albumin-bound fatty acids, rather than albumin alone, can induce OS and apoptosis in tubular cells [66, 67]. This effect can be enhanced by combining with FA to promote apoptosis in the proximal tubule [64].

In our study, analysis of immune cell infiltration revealed significant differences in the resting states of macrophages, mast cells, dendritic cells, and neutrophils between DN patients and the control group. Macrophages are the most common infiltrating immune cells in the kidneys of DN patients and are closely associated with renal function decline [68]. In DN, macrophages are closely linked to lipotoxicity, with studies showing macrophage infiltration surrounding lipotoxic renal tubular epithelial cells [69]. The role of macrophages in renal lipotoxicity is twofold. First, lipid accumulation in renal cells promotes the recruitment of macrophages, and second, lipotoxicity directly activates macrophages, leading to their differentiation and migration. Excessive and chronic uptake of lipids by macrophages is a contributing factor to the exacerbation of glomerular damage and atherosclerosis progression in patients with chronic kidney disease [10]. Our central gene and immune-related analysis results indicate a significant correlation between key genes related to lipotoxicity in DN and macrophages, consistent with the above findings. Mast cell infiltration and degranulation are closely related to the development of DN [70]. Mast cells are associated with angiogenesis, chronic inflammation, and fibrosis, with studies showing a significant increase in the number of mast cells in the glomeruli, interstitium, and perivascular areas of DN [71]. Mast cells release biologically active substances such as tryptase, chymase, TGF-*β*1, renin, and tumor necrosis factor *α* through degranulation into the renal tubulointerstitium, promoting kidney inflammation and fibrosis, thereby contributing to DN [72]. Neutrophil influx is associated with the acute response to inflammation or injury, but its contribution to the onset and progression of DN remains unclear. Evidence suggests that heightened spontaneous adhesion of neutrophils may play a role in this pathological process. However, it is important to note that this study is limited by its reliance on data from a public database. While we utilized another database to assess inflammation, additional experiments are necessary to validate these two biomarkers prior to their potential clinical application.

## 5. Conclusion

This study elucidates the fundamental mechanism underlying the onset and progression of DN. Enrichment analysis highlights the involvement of lipotoxicity-related pathways in disease advancement. Through the construction of a PPI network, two hub genes associated with lipotoxicity are identified as pivotal players in DN development. Furthermore, significant alterations in various immune cell populations during DN progression, closely linked to key LRGS, suggest that immune modulation can impact DN evolution. This study underscores the significance of investigating and comprehending the central LRGS lumican and ALB in DN, offering potential novel diagnostic biomarkers and laying a foundation for the development of drug targets related to lipotoxicity.

## Figures and Tables

**Table 1:** The detailed dataset information.

**Datasets**	**Platforms**	**Sample size**	**Organism**	**Tissue subregion**
GSE96804	GPL17586	41 DNs vs. 20 normal	*Homo sapiens*	Glomerulus
GSE104954	GPL24120	10 DNs vs. 5 normal	*Homo sapiens*	Tubulointerstitial

## Data Availability

The data used to support this study is available from the publicly available datasets, which can be received at https://www.ncbi.nlm.nih.gov/geo/.
